# Snack timing affects tissue clock and metabolic responses in male mice

**DOI:** 10.3389/fnut.2022.956641

**Published:** 2022-08-11

**Authors:** Kimberly Begemann, Henrik Oster

**Affiliations:** ^1^Institute of Neurobiology, University of Lübeck, Lübeck, Germany; ^2^Center of Brain, Behavior, and Metabolism, University of Lübeck, Lübeck, Germany

**Keywords:** caloric intake, circadian clock, body temperature, locomotor activity, snack

## Abstract

Snacking of small quantities of palatable food items throughout the day is common in modern societies and is promoted by 24/7 lifestyles. Long-term mistimed high-caloric food intake disrupts endogenous circadian rhythms and supports the development of obesity and other metabolic disorders. However, less is known about the time-of-day dependent effects of snacking. We hypothesized that already a single snacking episode may affect the circadian regulation of metabolic parameters, in particular when the snack is consumed during the daily rest phase. We performed an acute snack experiment in mice by providing access to chow or chocolate either at day- or nighttime and assessed snack effects on core body temperature, locomotor activity, and gene expression in metabolic tissues. Our results show that daytime chocolate snacking leads to a higher body temperature and locomotor activity increase compared to chow and nighttime intake. This goes along with altered clock and metabolic gene expression in peripheral tissues. Changes in nutrient uptake transporter gene expression in the small intestine suggest increased glucose resorption after daytime snacking. Our results indicate an early mechanism for the adipogenic effect of mistimed high-calorie snacking.

## Introduction

Eating palatable calorie-dense snacks during various phases of the day is a common behavior in Western societies with unlimited access to all kind of foods. A high-calorie diet is known to disrupt endogenous circadian rhythms and to shift food intake into the normal rest phase (1). While it is known that 1 week on a high-caloric diet is sufficient to shift endogenous rhythms in the liver (2), the acute effects of snacking on physiological parameters and circadian rhythms is much less understood.

Circadian clocks are internal timekeepers that synchronize physiology and behavior to external environmental rhythms such as the light-dark cycle (3). A master circadian pacemaker located in the suprachiasmatic nucleus (SCN) of the hypothalamus resets subordinate cellular clocks throughout the body to coordinate rhythmic functions within and between tissues and organs (4). At the molecular level, these clocks consist of interlocked transcriptional-translational feedback loops of clock proteins such as brain and muscle ARNT-like protein 1 (BMAL1 or ARNTL), circadian locomotor output cycles kaput (CLOCK), Period (PER1-3) and Nuclear receptor subfamily 1 group D member 1/2 (or NR1D1/2 or REVERBα/β) (3, 4).

While light is the main synchronizer of the SCN clock, altered food intake patterns can uncouple peripheral clocks from the SCN, thus leading to internal circadian desynchronization (5). In mice, daily food intake rhythms peak in the beginning of the active phase in line with the time of highest energy expenditure (6, 7). If food intake is restricted to the normal rest (i.e., light) phase energy expenditure is reduced and mice gain weight compared to animals with *ad libitum* food access (6). In contrast, appetite for highly palatable foods peak in the early inactive phase in mice, and also in humans, the drive to snack is higher toward the end of the active phase (i.e., in the evening) (7, 8). Sweet craving increases throughout the day in humans (9) and snacking rather than consuming one hot meal is common in night-shift workers (10). Studies on total energy intake in shift workers are controversial (11, 12), but disturbed eating patterns and increased snacking are consistently observed in night shift workers (13). Food intake-dependent signals such as insulin may provide feedback to the circadian system adjusting clock rhythms in metabolic tissues (14–16). However, the interaction of meal timing and type on the circadian clock system is still poorly understood.

We hypothesized that—in line with the increased vulnerability to snacking at this time—high-energy food intake would have stronger effects on circadian rhythms in the inactive phase. For this purpose, we offered mice chow or chocolate snacks at different times of day and investigated the impact on physiological parameters such as core body temperature and locomotor activity as well as on peripheral clock and clock target gene expression. Our data show that chocolate snacking has a stronger impact compared to chow on physiological parameters and tissue clock effects are largely restricted to the normal rest phase in mice.

## Materials and methods

### Animals and housing conditions

All experiments were performed with adult male wildtype mice on C57BL/6JRj background. Mice were purchased from Janvier labs (Le Genest-Saint-Isle, France). They were housed individually under standard laboratory conditions in a 12h: 12h light-dark cycle with *ad libitum* access to normal chow and tap water unless otherwise indicated. Experimental groups were age- and weight-matched. Experiments were carried out in accordance with the German Law for Animal Welfare and approved by the Ministry of Energy, Agriculture, Environment, Nature, and Digitalization (MELUND) of the State of Schleswig-Holstein, Germany.

### Core body temperature/locomotor activity recordings and acute snack experiment

All animals were implanted with G2 E-mitters (Starr Life Sciences, Oakmont, USA) into the intraperitoneal space to measure body temperature and activity. Mice were anesthetized with isoflurane (4% in air), injected with 4 mg/kg Carprofen (Rimadyl, Zoetis, Parsippany, USA) and Bepanthen (Bayer, Leverkusen, Germany) was applied on the eyes. The abdomen was shaved, disinfected and the abdominal cavity opened by first cutting the skin and then the muscular layer. The sterilized E-mitter was implanted and the two layers of the abdominal wall were closed separately. After 1 week of recovery, temperature and activity were recorded on the experimental day in 1-min intervals using ER4000 receivers (Starr Life Sciences) and the Vital View software, version 5 (Starr Life Sciences). On day four, mice were fasted for 12 h and then received a snack for 20 min (or nothing for the control cohort). Nighttime snack mice were fasted from *zeitgeber* time (ZT; ZT0 = light onset) 2/2.5 on day four onwards, receiving a snack at ZT14/14.5 whereas daytime snack mice fasted from ZT14/14.5 onwards and received a snack at ZT2/2.5 on the next day. Mice had access to the snack for 20 min. Control mice were fasted but received no snack. All animals were sacrificed after another 40 min (ZT3/3.5 or ZT15/15.5).

### Tissue and serum collection

Mice were sacrificed by cervical dislocation and trunk blood and tissues [subcutaneous white adipose tissue (scWAT), epididymal white adipose tissue (eWAT), intrascapular brown adipose tissue (iBAT), duodenum, jejunum, ileum, liver, pancreas] were collected. Tissue samples were stored in RNAlater (Invitrogen, ThermoFisher Scientific, Waltham, USA) and kept at 4°C for 11–12 h before transfer to storage at −20°C. Blood clotting was allowed at room temperature for 20 min followed by 30 min centrifugation at 664 rcf and 4°C. Serum samples were stored at −80°C until further processing.

### Food intake measurements

Food intake was determined for the 20-min re-feeding period on the experimental day. Snacks—chow [breeding diet #1314, Altromin, Lage, Germany (14% fat, 27% protein, 59% carbohydrates, metabolized energy: ~3,339 kcal/kg)] or chocolate (RUF milk chocolate drops, RUF, Quakenbrück, Germany; per 100 g: 2,099 kJ = 503 kcal, fat 27.1 g from which 16.7 g saturated fatty acids, carbohydrates 54 g from which 47.6 g sugar, protein 6.5 g)—were weighed before and after mice had access. It was confirmed for each mouse that food was not crumbled. Nutrient composition in chow and chocolate was calculated using the manufacturer's indication of food composition (Supplementary Figure 1).

### Serum levels of glucose, triacylglycerides, free fatty acids, insulin, and leptin

Serum glucose concentrations (mg/dL) were measured with a glucometer (ACCU-CHECK, Aviva, Roche, Mannheim, Germany). Triacylglyceride (TAG) concentrations were determined in duplicates using a triglyceride colorimetric assay kit (Cayman Chemical, Ann Arbor, USA) following the manufacturer's instructions. Free fatty acid (FFA) levels were measured in duplicates according to the kit's manual (serum/plasma fatty acid kit, non-esterified fatty acids detection, Zenbio, Durham, USA) with samples diluted 1:10 in dilution buffer. Serum insulin concentrations were measured in duplicates using a mouse insulin ELISA (Mercodia, Uppsala, Sweden) following the manufacturer's 5 μL protocol. A four-parameter logistic curve was fitted to calculate the concentrations. Due to limited material three samples were measured in singlets and the number of samples (*n* = 2–6) is not matching the *n* = 6 of glucose, FFA, and TAG. The blank absorbance value was subtracted from the mean of each duplicate and the concentration calculated according to the TAG, FFA or insulin standard curve, respectively. The insulin concentrations were converted as follows: 1 μg **≜** 174 pmol and 6 pmol/L **≜** 1 μIU/mL. Leptin levels were determined in duplicates using a mouse leptin ELISA kit (Crystal Chem, Elk Grove Village, USA) according to the kit's manual. Two samples were measured in singlets due to limited material. Data were analyzed in GraphPad Prism 8 (GraphPad, San Diego, USA).

### Quantitative real-time PCR

Total RNA was isolated from tissue homogenates (Omni Bead Ruptor 24, Omni International, Kennesaw, USA) by Trizol (Ambion, Life Technologies, Austin, USA) chloroform (≥99.5%, Honeywell, Charlotte, USA) extraction. RNA was reverse transcribed using the high-capacity cDNA reverse transcription kit (Applied Biosystems, Waltham, USA) according to the manufacturer's protocol. Go Taq qPCR master mix kit (Promega, Madison, USA) was used for qPCR. Plates were run on a CFX-96 or CFX-Connect thermocycler (Bio-Rad, Hercules, USA) and analyzed with the ΔΔC_t_ method using *Eef1*α as reference gene. Data were normalized to the mean ratio of the “no snack” group. Primer sequences were: *Bhlhe40* forward 5'-CTCCTACCCGAACATCTCAAAC-3', *Bhlhe40* reverse 5'-CCAGAACCACTGCTTTTTCC-3', *Bmal1* forward 5'-CCTAATTCTCAGGGCAGCAGAT-3', *Bmal1* reverse 5'-TCCAGTCTTGGCATCAATGAGT-3', *Clock* forward 5'-ATGGTGTTTACCGTAAGCTGTAG-3', *Clock* reverse 5'-CTCGCGTTACCAGGAAGCAT-3', *Cry2* forward 5'-AGATGGCCTCAGGTTTTCTCAG-3', *Cry2* reverse 5'-TTACGGCCCACTCTACCTTCT-3', *Eef1*α forward 5'- TGCCCCAGGACACAGAGACTTCA-3', *Eef1*α reverse 5'- AATTCACCAACACCAGCAGCAA-3', *Lipe* forward 5'-GGCTCACAGTTACCATCTCACC-3', *Lipe* reverse 5'- GAGTACCTTGCTGTCCTGTCC-3', *Per1* forward 5'-AGTTCCTGACCAAGCCTCGTTAG-3', *Per1* reverse 5'-CCTGCCCTCTGCTTGTCATC-3', *Per2* forward 5'-GCCAAGTTTGTGGAGTTCCTG-3', *Per2* reverse 5'-CTTGCACCTTGACCAGGTAGG-3', *Pnpla2* forward 5'-CAACGCCACTCACATCTACGG-3', *Pnpla2* reverse 5'-TCACCAGGTTGAAGGAGGGAT-3', *Nr1d1* forward 5'-AGCTCAACTCCCTGGCACTTAC-3', *Nr1d1* reverse 5'-CTTCTCGGAATGCATGTTGTTC-3', *Slc2a2* forward 5'-TCAGAAGACAAGATCACCGGA-3', *Slc2a2* reverse 5'-GCTGGTGTGACTGTAAGTGGG-3', *Slc5a1* forward 5'-TCTGTAGTGGCAAGGGGAAG-3', *Slc5a1* reverse 5'-ACAGGGCTTCTGTGTCTTGG-3'.

### Data and statistical analysis

Data are represented as group mean ± SEM. Statistical analyses were performed in GraphPad Prism 8 (GraphPad, San Diego, USA) whereby *p*-values < 0.05 were considered significant. To compare data between groups and different time points 2-way analysis of variance (ANOVA) was used with Bonferroni *post-hoc* tests to compare data at one ZT between groups or within one group at different ZT's, respectively.

Temperature and activity data were recorded in 1-min intervals. The 15 min before the snack was given to the mice were taken as baseline measures for temperature and activity. Mean temperature or activity were compared to the maximum change in body temperature or activity after “snack in.” Data were statistically analyzed by repeated measurement 2-way ANOVA with Bonferroni *post-hoc* tests. To further analyze changes in temperature and activity as well as the kinetics of these changes, each data point was normalized by subtracting the mean of the 15-min baseline. Such normalization allows a correction for individual differences in baseline body temperature and locomotor activity. For noise reduction in activity data a 10-min moving average was applied. Analyses were performed for each mouse individually. A non-linear fit (one site) was laid through the data for each group to determine the logIC50 value representing the half-time (in minutes) to the maximum change in temperature or activity. Analyses were performed with *n* = 6 mice for all cohorts; however, one no snack/daytime and one chow/nighttime animal were excluded due to technical reasons during the recordings. To calculate the mean change in temperature and activity, the mean of the 15 min before the snack was subtracted from the mean of the 1 h after “snack in” until sacrifice. To compare changes in temperature and activity (logIC50), non-linear fits were performed for each mouse individually. During fitting of temperature data, a constraint was set to 0 < logIC50 <60, but one no snack/nighttime and one chow/daytime mouse had to be excluded in addition to the two above mentioned mice because it was not possible to fit a sigmoidal curve with a plausible logIC50 through the data. For activity data the most accurate fits were obtained with a 10-min moving average over the non-normalized total counts. To not distort the fit due to decreasing activity in the second half of the experiment, only data including a moving average of 30.5 min were used to determine the half-time of activity change as a direct response to the snack. Constraints were set for some curves to ensure that the correct part of the curve was fitted compared to other mice in the group. In addition to the animals excluded for technical reasons, one no snack/nighttime mouse was excluded from activity logIC50 analysis as a reasonable fit could not be obtained. logIC50 values were statistically compared between cohorts and across time by 2-way ANOVA.

qPCRs were performed with *n* = 6 per cohort, but individual values were excluded based on Grubbs outlier tests. To analyze peripheral tissue gene expression profiles, the mean relative mRNA expression of the respective gene in every group was determined for each tissue, e.g., relative *Bmal1* mRNA expression in liver for the no snack/daytime group. Therefore, each data point in **Figure 4** represents one tissue. Heat maps how the mean gene relative mRNA expression for each tissue. To investigate the relationship between clock gene mRNA levels against glucose transporter gene expression, simple linear regressions were performed using GraphPad Prism 8 (GraphPad, San Diego, USA).

## Results

### Chocolate snack effects on caloric intake are higher during daytime

**Figure 1:**
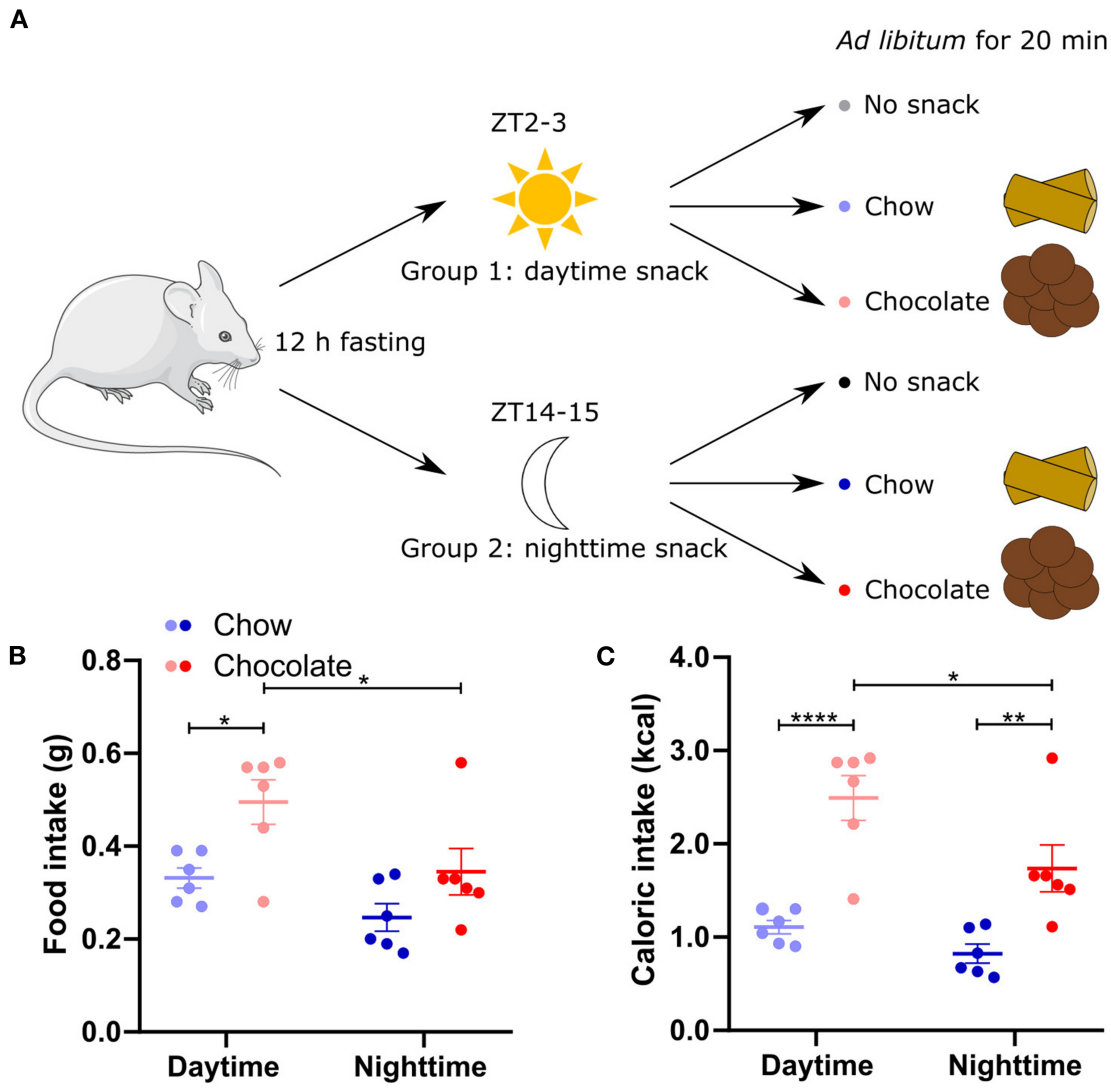
Food and caloric intake is higher after chocolate compared to chow snacking. **(A)** Experimental outline. Mice were separated in two groups to receive either a daytime (group 1) or nighttime (group 2) snack. After 12-h-fasting no snack (control cohort), normal chow, or chocolate was provided *ad libitum* as a snack for 20 min. **(B)** Food intake during 20 min chow or chocolate snacking. **(C)** Caloric intake during chow and chocolate snacking [calculated from **(B)**]. Data are shown as mean ± SEM; *n* = 6 per group; Bonferroni *post-hoc* test: *p < 0.05; **p < 0.01; ****p < 0.0001; 2-way ANOVA: **(B)** time, group *p* < 0.01, interaction *p* > 0.05, **(C)** time p < 0.05, group *p* < 0.0001, interaction *p* > 0.05. Mouse image: smart.servier.com.

To assess the time-dependent effect of different snacks, mice were separated into two groups of three cohorts each. After 12 h of fasting, they received either no snack (control cohorts), a chow snack (chow cohorts) or a chocolate snack (chocolate cohorts) with access for 20 min (Figure 1A). The first group received the snack (or no snack) in the beginning of the light phase (ZT2-3, daytime) whereas the second group received the snack (or no snack) in the beginning of the dark phase (ZT14-15, nighttime). Mice consumed more energy during daytime compared to nighttime snacking for chocolate but not for chow. Caloric intake was always higher in mice receiving the chocolate compared to the chow snack independent of snack timing (Figures 1B,C). These data suggest that both, snack type and timing, influence appetite regulation, but temporal regulation is more pronounced for more palatable snack types.

### Snack type dependent upregulation of core body temperature and locomotor activity is restricted to daytime

**Figure 2:**
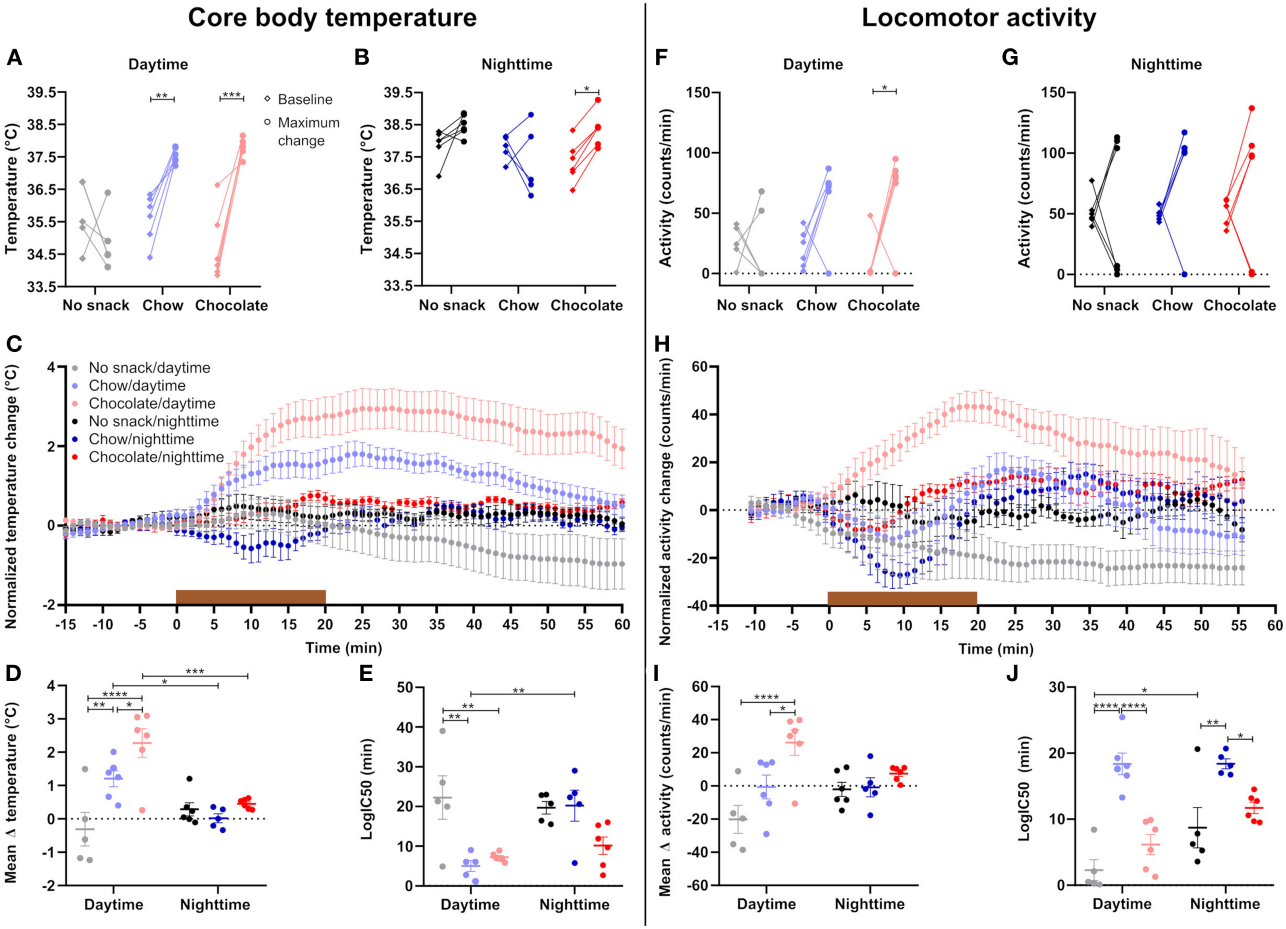
Daytime snacking leads to temperature and locomotor activity upregulation with a step-wise increase in chow and chocolate. Mean body temperature during the 15 min baseline (♢, left) and maximum change (∘, right) in body temperature upon **(A)** daytime and **(B)** nighttime snacking for each mouse. **(C)** Change in body temperature upon snacking. Data were normalized by subtracting the mean temperature during the 15 min baseline. **(D)** Mean change in body temperature. **(E)** logIC50 from individual non-linear fits of body temperature curves. Mean activity during the 15 min baseline (♢, left) and maximum change (∘, right) in activity upon **(F)** daytime and **(G)** nighttime snacking for each mouse. **(H)** Change in activity shown as 10-min moving average. Data were normalized by subtracting the mean activity during the 15 min baseline. **(I)** Mean change in activity. **(J)** logIC50 from individual non-linear fits of activity curves. Data are shown as mean ± SEM; *n* = 5–6 per group; **(A,B)** Bonferroni *post-hoc* test: *p < 0.05; **p < 0.01; ***p < 0.001; **(A)** Maximum change no snack vs. chow *p* < 0.0001, maximum change no snack vs. chocolate *p* < 0.0001; **(B)** Maximum change no snack vs. chow *p* < 0.05; Repeated measurement 2-way ANOVA: **(A)** time, group, interaction *p* < 0.001; **(B)** time, group *p* > 0.05, interaction *p* < 0.05; **(C,H)** Non-linear fit. Brown bar indicates snack access for 20 min. **(D,E,I,J)** Bonferroni *post-hoc* test: *p < 0.05; **p < 0.01; ***p < 0.001; ****p < 0.0001; 2-way ANOVA: **(D)** time *p* < 0.01, group *p* < 0.001, interaction, *p* < 0.01, **(E)** time *p* < 0.05, group *p* < 0.001, interaction *p* < 0.05; **(I)** time *p* > 0.05, group *p* < 0.001, interaction *p* < 0.05, **(J)** time *p* < 0.01, group *p* < 0.0001, interaction *p* > 0.05. **(F,G)** Bonferroni *post-hoc* test: *p < 0.05; Repeated measurement 2-way ANOVA: **(F)** group *p* < 0.05, time, interaction *p* > 0.05; **(G)** time, group, interaction *p* > 0.05.

Food intake leads to a transient increase in body temperature and locomotor activity, and both have been implicated in the postprandial regulation of satiety (17, 18). We thus examined the interaction of time and snack type on these two physiological parameters. After daytime snacking body temperature significantly increased compared to baseline in the chow and chocolate cohorts (Figure 2A) whereas a temperature increase after nighttime snacking was only observed in the chocolate cohort (Figure 2B). We next investigated the kinetics of the temperature increase upon snacking. While body temperature increased comparably early upon, both, chow and chocolate snacking during daytime, this increase was higher, and temperature stayed elevated for longer in the chocolate cohort (Figure 2C). In comparison, body temperature in the chow/daytime snacking cohort dropped back to baseline levels 1 h after the snack was provided (Figure 2C). In contrast to daytime, body temperature changes in the nighttime cohorts were smaller and largely comparable between snack types (Figures 2C,D). We did not observe differences in temperature increases between the control cohorts at different ZTs, but we found much larger temperature effects in the chow/daytime and chocolate/daytime compared to the respective nighttime cohorts (Figure 2D). Daytime snacking led to a stronger increase in body temperature in the chocolate cohort compared to chow (and no snack) with mean temperature changes of 2.6°C, 1.5°C and −0.3°C, respectively (Figure 2D). To compare the kinetics of body temperature changes upon snacking we performed a non-linear fit for each individual curve and determined logIC50 values. Daytime snacking with chow and chocolate induced a fast increase in body temperature (logIC50 <10 min; Figure 2E). Additionally, chow/daytime snacking induced a faster body temperature increase than in the chow/nighttime cohort (logIC50 of 5 and 20 min, respectively; Figure 2E). These temporal effects were much less pronounced for the chocolate snack cohorts [logIC50 values of 7 (daytime) and 10 min (nighttime); Figure 2E]. Together, these data suggest that daytime snacking induces larger changes in body temperature and that these changes differ for snack type.

Similar to what we observed for body temperature, locomotor activity was affected mostly by snacking during daytime, while little effects were observed at nighttime (Figures 2F–H). Significant increases in locomotor activity compared to baseline activity were restricted to the chocolate/daytime cohort (Figures 2F,G). While the no snack/daytime cohort became less active due to extended fasting, chow-fed animals showed on average stable activity and chocolate/daytime mice a marked increase in activity peaking at 20 min after snack access (Figures 2H,I). Individual non-linear curve fits revealed delayed changes in activity in the chow cohorts at both time points compared to control and chocolate fed animals (Figure 2J). Overall, we observed a time-of-day effect for activity kinetics with a significant *post-hoc* comparison for the control cohorts (Figure 2J). In summary, the extents of locomotor activity changes upon snacking are in line with body temperature effects with larger changes induced by daytime snacking and chocolate snacks. However, marked differential effects were observed with regard to kinetics suggestive of distinct regulatory mechanisms.

### Daytime snacking alters serum glucose and free fatty acid levels

**Figure 3:**
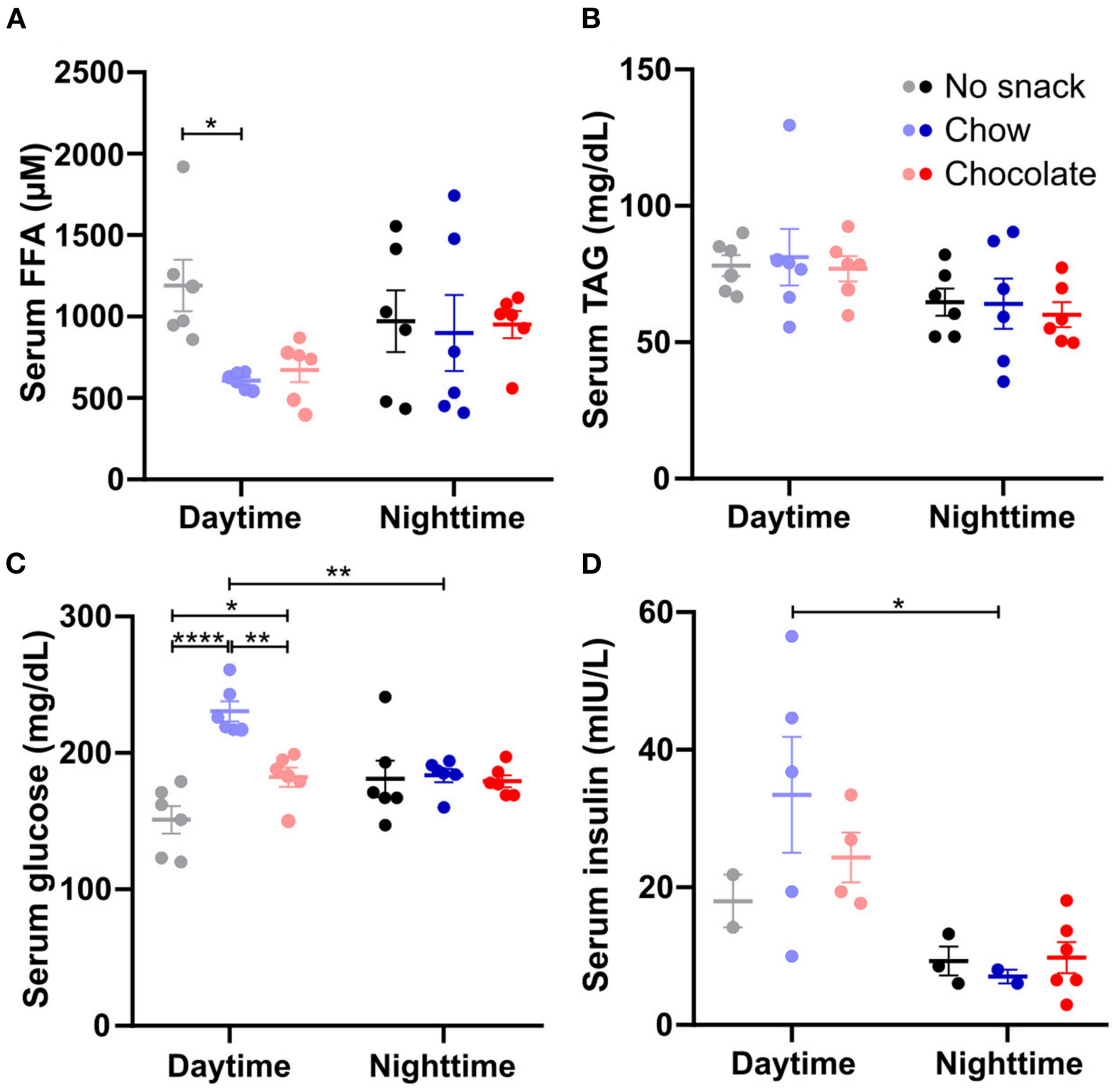
Daytime snacking alters serum free fatty acid (FFA) and glucose levels. Concentrations of **(A)** FFAs, **(B)** triacylglycerides (TAGs), **(C)** glucose, and **(D)** insulin in serum. Mice were fasted for 12 h and received no snack (control cohort), normal chow, or chocolate *ad libitum* for 20 min. Data are shown as mean ± SEM; **(A–C)** *n* = 6, **(D)** *n* = 2–6 per group; Bonferroni *post-hoc* test: *p < 0.05; **p < 0.01; ****p < 0.0001; 2-way ANOVA: **(A)** time, group, interaction *p* > 0.05, **(B)** time *p* < 0.01, group, interaction *p* > 0.05, **(C)** time *p* > 0.05, group, interaction *p* < 0.001, **(D)** time *p* < 0.01, group, interaction *p* > 0.05.

We next analyzed the impact of timed chow or chocolate snacking on postprandial nutrient levels in the circulation (Figure 3). 1 h after the snack serum FFA levels were significantly decreased in the chow/daytime cohort and reduced with borderline significance (*p* = 0.0540) after chocolate snacking during the day compared to no snack controls (Figure 3A). Serum FFA concentrations were not altered in the nighttime cohorts (Figure 3A). We did not observe any snack induced changes in serum TAG levels apart from a general time effect with slightly higher levels at daytime compared to nighttime (Figure 3B). Serum glucose concentrations were increased after chow and chocolate daytime snacking compared to controls with higher levels in the chow cohort (Figure 3C). Nighttime serum glucose concentrations did not differ between the cohorts. Serum insulin levels were higher at daytime compared to nighttime, but we did not observe snack induced differences (Figure 3D). However, the insulin concentrations roughly followed serum glucose concentrations (Figures 3C,D). We did neither observe a significant effect for time nor snack group in serum leptin levels (Supplementary Figure 2). Together, snacking decreased FFA and increased glucose serum levels specifically during the day hinting at temporal differences in lipid mobilization and glucose uptake.

### Time and snack dependent regulation of clock and metabolic gene expression in peripheral tissues

**Figure 4:**
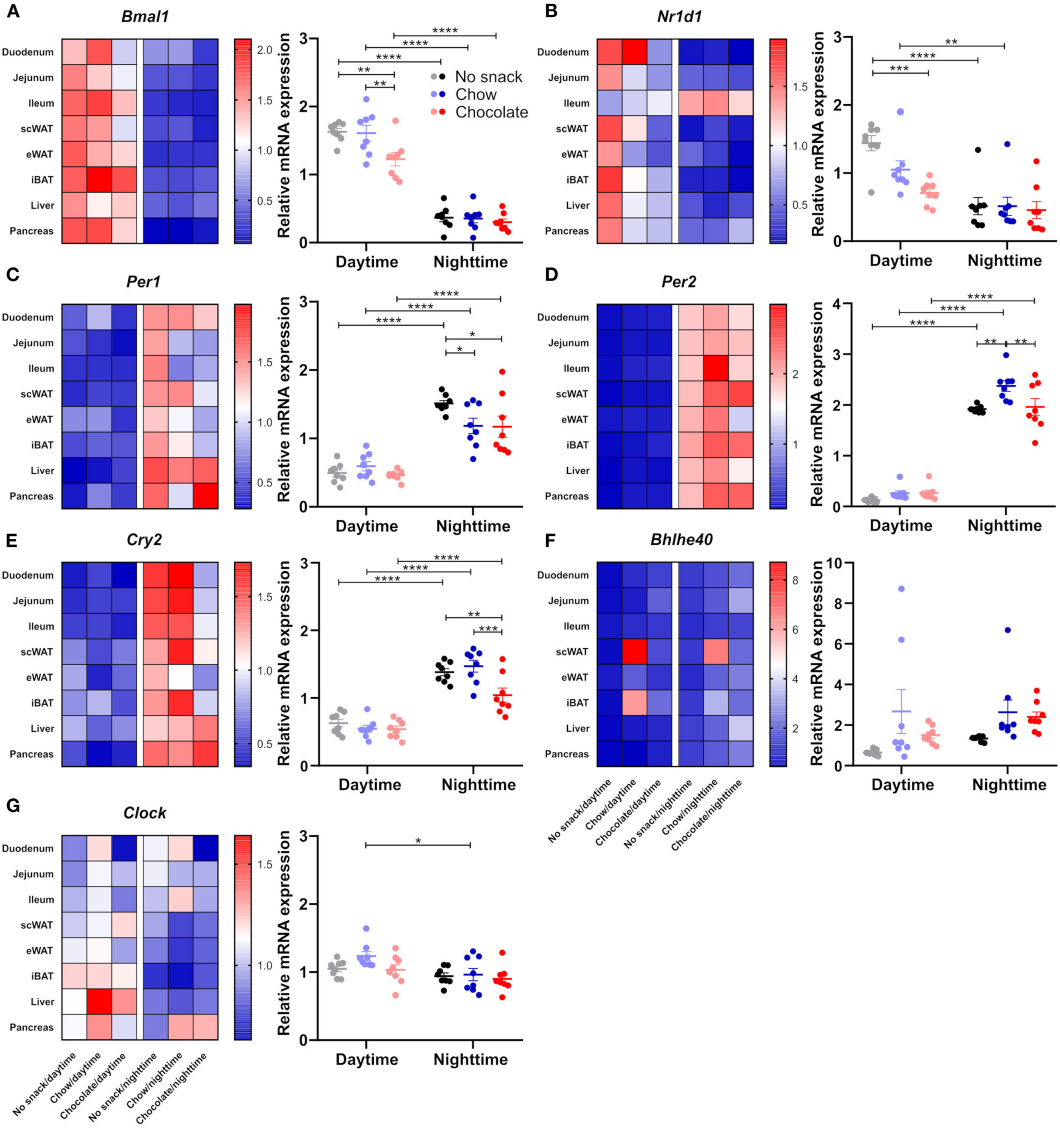
Snacking affects clock gene expression in peripheral tissues. Mice were fasted for 12 h and received no snack (control cohort), normal chow, or chocolate *ad libitum* for 20 min either at daytime or nighttime. Clock gene expression was determined 1 h after the snack was provided. Heat map of mean gene relative mRNA expression for each tissue and mean gene relative mRNA expression of **(A)** *Bmal1*, **(B)** *Nr1d1*, **(C)** *Per1*, **(D)** *Per2*, **(E)** *Cry2*, **(F)** *Bhlhe40*, **(G)** *Clock* in peripheral tissues (duodenum, jejunum, ileum, subcutaneous and epididymal white adipose tissue, intrascalpular brown adipose tissue, liver, pancreas). Each data point represents one tissue. Data are shown as mean ± SEM; *n* = 3–6 within *n* = 8 tissues per group; Bonferroni *post-hoc* test: *p < 0.05; **p < 0.01; ***p < 0.001; ****p < 0.0001; 2-way ANOVA: **(A)** time *p* < 0.0001, group *p* < 0.01, interaction *p* > 0.05; **(B)** time *p* < 0.0001, group *p* < 0.01, interaction *p* < 0.05; **(C)** time *p* < 0.0001, group, interaction *p* > 0.05; **(D)** time *p* < 0.05, group *p* < 0.01, interaction *p* < 0.05; **(E)** time *p* < 0.0001, group *p* < 0.01, interaction *p* < 0.05; **(F)** group *p* < 0.05, time, interaction *p* > 0.05; **(G)** time *p* < 0.01, group, interaction *p* > 0.05. Separate analysis of each gene in each tissue as well as statistics are provided in Supplementary Figure 3.

Considering the observed time and snack type specific responses in body temperature as well as in locomotor activity against a background of circadian regulation of both processes, we hypothesized that tissue clock gene expression might likewise be affected by snacking with higher changes after chocolate snacking and during daytime. We focused on peripheral tissues important for the processing of nutrients such as liver, pancreas, adipose tissues (eWAT, scWAT, and iBAT), and small intestine (duodenum, jejunum, and ileum). Overall, results were largely comparable across all these tissues with marked temporal differences for *Bmal1, Per1, Per2, Cry2* and *Nr1d1* in the control cohorts and snack effects confined mostly to *Bmal1* and *Nr1d1* expression during daytime and *Per1, Per2* and Cry2 expression during nighttime (Supplementary Figure 3). To provide a more general overview, we plotted a heat map showing the mean relative mRNA expression for the respective gene and determined specific clock gene responses across metabolic tissues by averaging the mean relative clock gene mRNA expression for each group (Figure 4). A detailed analysis of each single gene in each tissue is provided in Supplementary Figure 3. Across all tissues, *Bmal1* expression was higher at daytime compared to nighttime with antiphasic regulation of *Per1, Per2*, and *Cry2* in the periphery in all snack cohorts (Figures 4A,C–E). *Bmal1* expression was decreased at daytime after chocolate but not after chow snacking while levels were stable during nighttime (Figure 4A). In contrast, *Per1* expression was decreased in the chow and chocolate cohorts during nighttime snacking with no effect during daytime (Figure 4C). For *Nr1d1*—and similar to *Bmal1*—we observed a snack induced decrease in expression during daytime, but no snack effect in the night (Figure 4B). Effects on *Per2* and *Cry2* expression were restricted to nighttime (Figures 4D,E). *Per2* was increased in the chow/nighttime cohort compared to the control and chocolate cohorts (Figure 4D). *Cry2* was decreased after chocolate/nighttime snacking compared to the chow and nighttime cohort (Figure 4E). *Bhlhe40* expression was comparable between different tissues at day-/nighttime after different snacks, however, it was increased in scWAT in the chow cohorts as well as in iBAT after chow/daytime snacking (Figure 4F). *Clock* mRNA expression did not respond to snack intake, but slightly higher daytime mRNA levels compared to nighttime were observed in the chow cohorts (Figure 4G). In summary, time and snack type dependent effects on clock gene mRNA expression were observed in metabolic tissues. Regulation of *Bmal1* and *Nr1d1* was restricted to daytime, while *Per1, Per2*, and *Cry2* responses were seen only after nighttime snacking.

**Figure 5:**
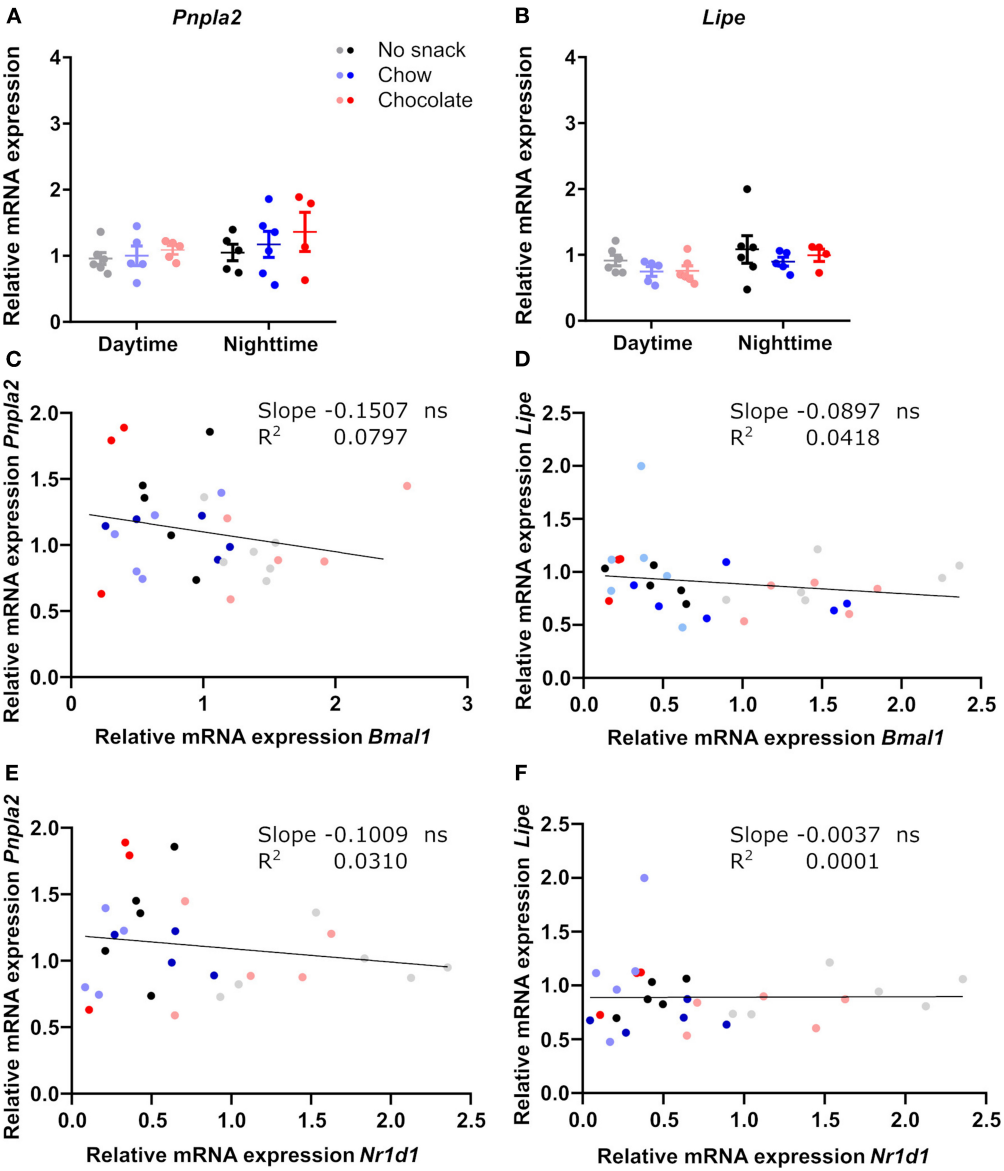
Genes of lipolysis key-enzymes in scWAT are not affected by snacking and do not correlate with clock gene expression. Relative mRNA expression of **(A)** *Pnpla2*, **(B)** *Lipe*. Linear regression of **(C)** *Bmal1* and *Pnpla2*, **(D)** *Bmal1* and *Lipe*, **(E)** *Nr1d1* and *Pnpla2*, **(F)** *Nr1d1* and *Lipe*. **(A,B)** Data are shown as mean ± SEM; *n* = 4–6 per group; 2-way ANOVA (time, group, interaction *p* > 0.05) with Bonferroni *post-hoc* test. **(C–F)** Simple linear regression analysis; *n* = 3–6 per group. ns, not significant.

Reduced FFA levels after snacking during daytime suggested an effect on lipolysis in adipose stores (Figure 3A). We therefore compared responses of clock and lipolysis associated gene expression in scWAT (Supplementary Figure 3, Figure 5). *Bmal1* expression was higher at daytime compared to nighttime for all groups and was reduced after chocolate/daytime snacking compared to the control cohort (Supplementary Figure 3D). We observed similar effects for *Nr1d1* expression, but the time-of-day difference between the chocolate cohorts was lost due to a strong reduction in *Nr1d1* expression after chocolate/daytime snacking (Supplementary Figure 3D). Additionally, *Nr1d1* expression was reduced in the chocolate compared to the chow/daytime cohort (Supplementary Figure 3D). In contrast, we did not find any changes in genes encoding two lipolysis pacemaker enzymes, *Pnpla2* and *Lipe* (Figures 5A,B), nor a correlation between clock and lipolysis associated gene expression (Figures 5C–F). Thus, although FFA serum levels were reduced after daytime snacking (Figure 3A) and lipolysis genes have been reported to be under control of the circadian clock (19), our data would be in line with a snack-induced downregulation of adipose lipolysis *via* resetting of local tissue clocks.

**Figure 6:**
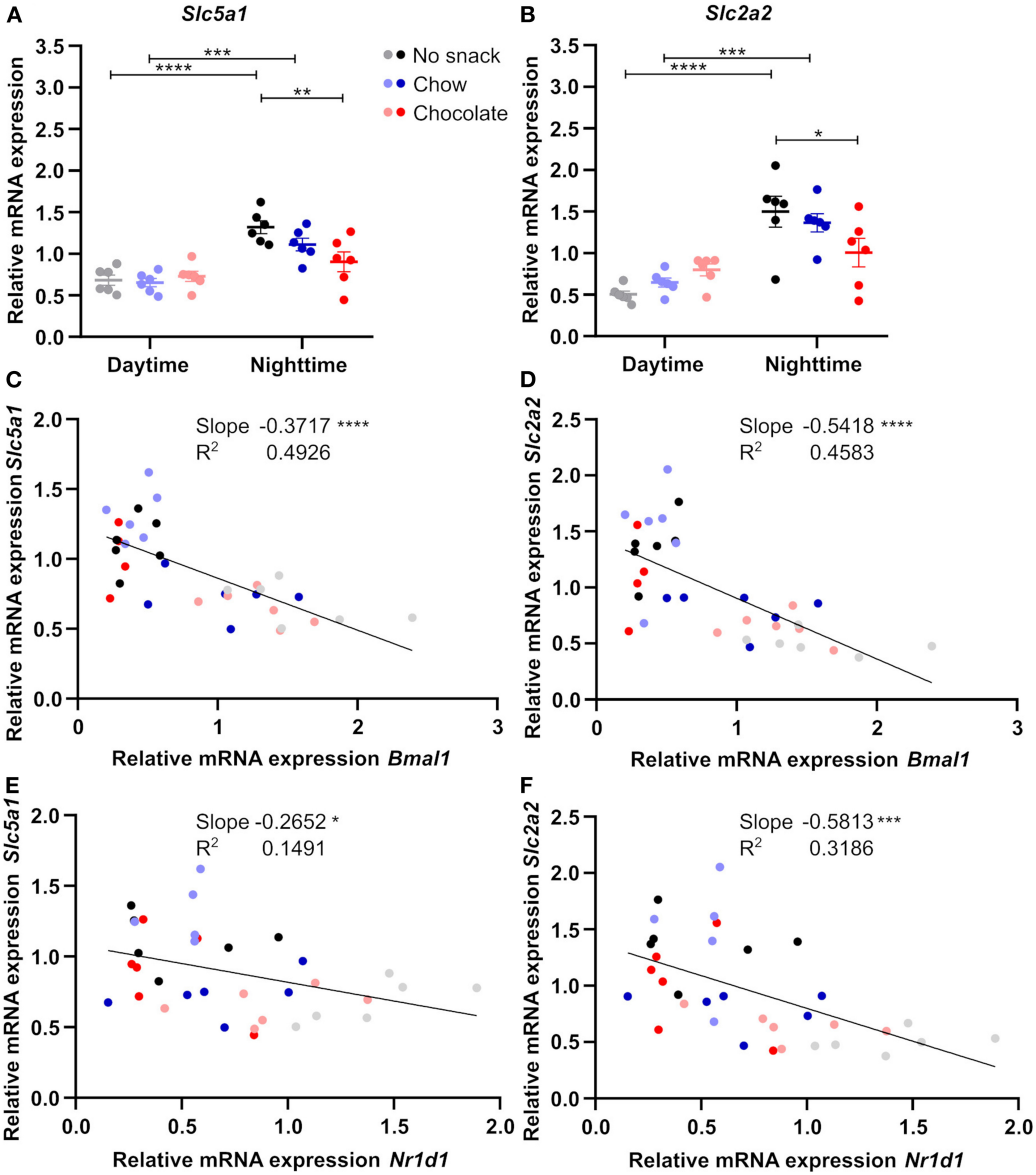
Jejunal glucose uptake transporter gene expression is influenced by snack type and negatively correlates with clock gene expression. Relative mRNA expression of **(A)** *Slc5a1*, **(B)** *Slc2a2*. Linear regression of **(C)** *Bmal1* and *Slc5a1*, **(D)** *Bmal1* and *Slc2a2*, **(E)** *Nr1d1* and *Slc5a1*, **(F)** *Nr1d1* and *Slc2a2*. **(A,B)** Data are shown as mean ± SEM; *n* = 5–6 per group; Bonferroni *post-hoc* test: *p < 0.05; **p < 0.01; ***p < 0.001; ****p < 0.0001; 2-way ANOVA **(A)** time *p* < 0.0001, group *p* > 0.05, interaction *p* < 0.05, **(B)** time *p* < 0.0001, group *p* > 0.05, interaction *p* < 0.01. **(C–F)** Simple linear regression analysis; *n* = 4–6 per group. *p < 0.05; ***p < 0.001; ****p < 0.0001.

Like FFAs, blood glucose levels were mainly affected by snack intake during daytime. As the small intestine, especially duodenum and jejunum, are important for glucose uptake (20), we investigated relative mRNA expression of glucose transporter genes in comparison to clock genes in response to snack intake (Supplementary Figure 3, Figure 6). Expression of *Bmal1* and *Nr1d1* in the jejunum was reduced after snacking during daytime with little responses during nighttime snacking (Supplementary Figure 3B). The expression of both glucose uptake transporter genes, *Slc5a1* and *Slc2a2*, was decreased in the chocolate/nighttime cohort compared to the no snack/nighttime cohort leading to a loss of the time-of-day difference in chocolate cohorts (Figures 6A,B). As both, clock and glucose uptake transporter, transcript levels were affected by snacking, we investigated the relationship between both by linear regression analysis. Interestingly, we found a negative correlation between *Bmal1* and *Nr1d1*, respectively, with both glucose uptake transporter genes (Figures 6C–F).

Duodenal clock gene expression was reduced after chocolate/daytime snacking compared to the chow cohort (*Bmal1*) or the control and chow cohorts (*Nr1d1*), respectively (Supplementary Figure 3A). All cohorts had a higher expression at daytime compared to nighttime except of the chocolate cohort for *Nr1d1* (Supplementary Figure 3A). Interestingly, relative mRNA expression of glucose uptake transporters was decreased in the chocolate cohorts at both time points compared to the control and chow cohorts except of control vs. chocolate/daytime for *Slc5a1* (Supplementary Figures 4A,B). However, unlike for jejunum, we did not find significant correlations between clock and glucose transporter gene expression in the duodenum (Supplementary Figures 4C–F).

The observed relationship of clock and glucose uptake transporter gene expression in the jejunum, but not in the duodenum, support the idea of a potential role of the jejunal circadian clock in mediating changes in intestinal glucose uptake upon snacking.

## Discussion

In our study we investigated time-of-day dependent effects of snacking on tissue circadian clock function and physiological parameters. Our results indicate that a high-calorie snack such as chocolate at daytime has a stronger effect on physiological parameters as well as on peripheral clock gene expression than less energy-dense snack types or the same snack consumed during nighttime. This temporal regulation associates with similar effects on glucose regulation in line with clock and glucose uptake transporter gene regulation in the jejunum.

### Snack type and time influence appetite and temperature/activity responses

In our snack experiment we observed an effect of snack type and snack time on behavioral and physiological responses. Chocolate consumption was always higher than chow consumption and chocolate/daytime mice consumed more than the respective nighttime cohort (Figure 1). These results go in line with data showing that overconsumption of chocolate peak in the early inactive phase in mice and time-of-day differences in the drive to eat in humans (7, 8). Likewise, we found a snack type dependent upregulation of core body temperature and locomotor activity mostly restricted to daytime (Figure 2). As body temperature shows a circadian rhythm with higher temperature throughout the active phase (21) and even on heat stress mice show little increase in body temperature throughout their active phase (22), a large body temperature increase upon snacking was not expected at nighttime. We observed a higher upregulation in body temperature after chocolate compared to chow/daytime snacking. As mice consumed more calories after a chocolate snack, these findings are in line with previous studies showing that body temperature is higher after high caloric meals (23). It has previously been shown that diet composition impacts body temperature (24). Considering the different nutrient composition of the snacks in this study (Supplementary Figure 1), an effect of snack type on body temperature change remains possible. However, we did not closer investigate the specific effects of individual macronutrients and therefore cannot distinguish which factor—snack type (i.e. snack composition) or calories—is mediating the observed effects. In line with the changes in core body temperature we found similar changes in locomotor activity after daytime snacking. Larger changes throughout nighttime snacking were not expected as the capacity for changes is higher throughout the usual inactive phase (i.e., daytime). Overall, we did not observe a mean change in activity in chow snacking mice. These animals first reduced their activity as they were eating the snack and then became more active afterwards. Our observation of upregulated locomotor activity after chocolate/daytime snacking is in line with human data showing that a daily chocolate snack for dinner leads to increased activity (25). However, one has to consider that subjects were monitored after a chronic chocolate snack showing overall activity (25). Additionally, our data of the chow/daytime cohort show that temperature increases do not merely follow upregulated locomotor activity.

### Clock gene responses are influenced by snack time and type

Because of our findings of upregulated core body temperature and locomotor activity restricted to daytime snacking, we assumed a snack effect on peripheral clock gene expression. We found slightly more reduced relative mRNA expression of clock genes in the periphery after chocolate/daytime snacking hinting at a potential influence of snack type on clock gene expression (Figure 4). After nighttime snacking, we observed changes in the chow as well as in the chocolate cohorts (Figure 4). Clock gene expression is shifting and uncoupling from the SCN upon light-phase restricted feeding (5). But also fasting and re-feeding is changing peripheral tissue clock gene expression (26). Although our data do not determine full circadian profiles, they are in line with others showing downregulation of *Nr1d1* and *Per1* mRNA expression upon re-feeding (26). *Bmal1* mRNA expression was rather seen to be upregulated (26). The differences of those results and ours could be explained by different re-feeding times, durations and snack types. As we investigated only two time points, we cannot comment on circadian rhythm parameters such as period, phase or amplitude.

### A potential role of jejunal circadian clocks in regulation of glucose metabolism and effects of snack type and timing

Our results revealed decreased FFA and increased glucose serum levels specifically in response to daytime snacking (Figure 3) suggesting alterations in lipolysis and glucose uptake. Upon fasting, lipolysis is enhanced increasing FFA levels in the blood (27). After daytime re-feeding in rats, FFA concentrations in serum decreases again in line with our data (27). Another study found a fast reduction of FFA upon food intake in humans after breakfast and dinner (28). We did not observe decreased serum FFA levels after nighttime snacking in mice. However, these animals were only fasted for 12 h, so basically throughout daytime, the usual phase where they normally do not consume many calories. Potentially, the difference in FFA levels upon snacking was therefore more visible after daytime snacking because these cohorts were likely fasted even longer than 12 h due to the normal daily food intake pattern with low intake during the light phase. Our observation of unchanged TAG levels after daytime or nighttime snacking are in line with other data showing that plasma TAG levels in humans are unaltered after breakfast and dinner at least 1 h after the meal (28). The apparent absence of changes in leptin levels (Supplementary Figure 2) could be explained by different kinetics of leptin responses after snacking. We found altered serum glucose levels after daytime snacking and a time-of-day difference after chow snacking. These results are consistent with data showing that glucose tolerance varies throughout the day peaking in the morning (active phase) and that the postprandial glucose response is higher in the evening in humans (29). A higher increase in glucose concentration is also consistent with human data showing a higher glucose increase after dinner vs. breakfast (28). Unexpectedly, glucose levels after chocolate/daytime were lower compared to chow/daytime snacking. Mice consumed the chocolate within the very first min after the snack was provided which could indicate that the glucose levels were already dropping again, but we also cannot exclude an influence of snack type/food composition on serum parameters. Glucose uptake by peripheral tissues could be differently affected in the two daytime snacking cohorts. More glucose could be taken up by adipose tissue, by the liver for glycogen storage, or by the muscle and thereby reduce serum glucose levels in the chocolate/daytime cohort. As mice consume most of their food during the active phase, equal experimental fasting will likely be longer for the daytime than for the nighttime cohorts and, consequently, daytime cohorts might have been at a different fasting/metabolic state in the beginning of the snack time. Differences in insulin release upon chow or chocolate consumption could also explain the observed changes in the chow/daytime and chocolate/daytime cohorts. In our study insulin concentrations followed changes in glucose, however, they did not reach significance due to high variations within the cohorts. Lower insulin levels in the nighttime cohorts could indicate a higher insulin sensitivity at nighttime compared to daytime. A higher insulin sensitivity in the beginning of the active phase was also reported in humans (30). Additionally, changes in lipolysis are a possible explanation for the differences we observed in FFA serum concentrations after daytime snacking.

Due to the downregulation of *Bmal1* expression after chocolate, but not after chow/daytime snacking, we analyzed lipolysis related gene expression and the relationship of lipolysis and clock gene transcript levels. *Pnpla2* and *Lipe* are downregulated in *Bmal1* knockout mice (19). However, our data did not support a downregulation of lipolysis in scWAT upon daytime snacking (Figure 5). FFA are increased in the blood upon fasting and decrease after re-feeding (27). We already saw decreased FFA levels upon daytime snacking (Figure 3). However, the analyzed time point might be too early to observe changes upon lipolysis related genes or the effect of fasting might be stronger on mRNA expression. We also did not observe a relationship between clock and lipolysis related gene expression.

Our results indicate changes in clock and glucose uptake transporter gene expression upon snacking in the small intestine (Figure 6, Supplementary Figures 3, 4). Studies in cells revealed a regulation of BMAL1 on glucose uptake and *Slc5a1* mRNA expression (31). Additionally, BMAL1 can directly bind to the promotor region of glucose uptake transporter genes (32). In line with these data, we found an association between clock and glucose uptake transporter gene expression in the jejunum. Importantly, we mainly observed changes in glucose uptake transporter gene expression upon chocolate snacking. Even though the chocolate contains less carbohydrates compared to chow (Supplementary Figure 1), it contains more disaccharides (carbohydrates 54 g from which 47.6 g sugar vs. 5% dissaccharides but 35% polysaccharides in normal chow). Our data, thus, are in agreement with a potential role of the jejunal circadian clock on glucose uptake upon snacking.

In conclusion, we here show that distinct snack types have different effects on tissue circadian clocks whereby the effect is strongest after snacking during daytime. Our data suggest that an acute snack could influence the circadian clock and modulate glucose uptake in the small intestine. With our experimental setup we analyzed acute snack effects on gene expression, but we cannot make conclusions about how these effects would alter circadian rhythm parameters. As our study is limited to male mice it would be interesting to investigate whether the same effect is observed in females. These observations in mice could be interesting for shift workers with an irregular eating pattern. Although our data suggest a potential role of the jejunal clock in snack associated glucose uptake, further confirmatory work is needed to, e.g., investigate whether an acute snack at the “wrong” time of the day could lead to a larger shift in the internal clock network.

## Data availability statement

The raw data supporting the conclusions of this article will be made available by the authors, without undue reservation.

## Ethics statement

The animal study was reviewed and approved by the Ethics Committee of the Ministry of Energy, Agriculture, Environment, Nature, and Digitalization (MELUND) of the State of Schleswig-Holstein, Germany.

## Author contributions

KB and HO designed the experiments, wrote the paper, revised, and approved the submitted version. KB performed experiments and analyzed data. All authors contributed to the article and approved the submitted version.

## Funding

This publication was supported by a grant of the German Research Foundation (DFG) to HO: RTG-1957 Adipocyte-Brain Crosstalk. The funder was not involved in the study design, collection, analysis, interpretation of data, the writing of this article or the decision to submit it for publication.

## Conflict of interest

The authors declare that the research was conducted in the absence of any commercial or financial relationships that could be construed as a potential conflict of interest.

## Publisher's note

All claims expressed in this article are solely those of the authors and do not necessarily represent those of their affiliated organizations, or those of the publisher, the editors and the reviewers. Any product that may be evaluated in this article, or claim that may be made by its manufacturer, is not guaranteed or endorsed by the publisher.

## References

[1] Kohsaka A Laposky AD Ramsey KM Estrada C Joshu C Kobayashi Y. High-fat diet disrupts behavioral and molecular circadian rhythms in mice. Cell Metab. (2007) 6:414–21. 10.1016/j.cmet.2007.09.006 17983587

[2] Pendergast JS Branecky KL Yang W Ellacott KLJ Niswender KD Yamazaki S. High-fat diet acutely affects circadian organisation and eating behavior. Eur J Neurosci. (2013) 37:1350–6. 10.1111/ejn.12133 23331763 PMC3645495

[3] Partch CL Green CB Takahashi JS. Molecular architecture of the mammalian circadian clock. Trends Cell Biol. (2014) 24:90–9. 10.1016/j.tcb.2013.07.002 23916625 PMC3946763

[4] Buhr ED Takahashi JS. Molecular components of the mammalian circadian clock. In: Kramer A, Merrow M, editors. Circadian Clocks. Handbook of Experimental Pharmacology, Vol. 217. Berlin; Heidelberg: Springer (2013). p. 3–27. 10.1007/978-3-642-25950-0_1 PMC3762864 23604473

[5] Damiola F Le Minh N Preitner N Kornmann B Fleury-Olela F Schibler U. Restricted feeding uncouples circadian oscillators in peripheral tissues from the central pacemaker in the suprachiasmatic nucleus. Genes Dev. (2000) 14:2950–61. 10.1101/gad.183500 11114885 PMC317100

[6] Bray MS Ratcliffe WF Grenett MH Brewer RA Gamble KL Young ME. Quantitative analysis of light-phase restricted feeding reveals metabolic dyssynchrony in mice. Int J Obes. (2013) 37:843–52. 10.1038/ijo.2012.137 22907695 PMC3505273

[7] Koch CE Begemann K Kiehn JT Griewahn L Mauer J M E Hess null. Circadian regulation of hedonic appetite in mice by clocks in dopaminergic neurons of the VTA. Nat Commun. (2020) 11:3071. 10.1038/s41467-020-16882-6 32555162 PMC7299974

[8] Chamorro R Kannenberg S Wilms B Kleinerüschkamp C Meyhöfer S Park SQ. Meal timing and macronutrient composition modulate human metabolism and reward-related drive to eat. Nutrients. (2022) 14:562. 10.3390/nu14030562 35276920 PMC8839823

[9] Reichenberger J Richard A Smyth JM Fischer D Pollatos O Blechert J. It's craving time: time of day effects on momentary hunger and food craving in daily life. Nutrition. (2018) 55–56:15–20. 10.1016/j.nut.2018.03.048 29960151

[10] Waterhouse J Buckley P Edwards B Reilly T. Measurement of, and some reasons for, differences in eating habits between night and day workers. Chronobiol Int. (2003) 20:1075–92. 10.1081/cbi-120025536 14680144

[11] Lennernäs M Hambraeus L Akerstedt T. Shift related dietary intake in day and shift workers. Appetite. (1995) 25:253–65. 10.1006/appe.1995.0060 8746965

[12] Hulsegge G Boer JM van der Beek AJ Verschuren WM Sluijs I Vermeulen R. Shift workers have a similar diet quality but higher energy intake than day workers. Scand J Work Environ Health. (2016) 42:459–68. 10.5271/sjweh.3593 27631649

[13] Reeves SL Newling-Ward E Gissane C. The effect of shift-work on food intake and eating habits. Nutr Food Sci. (2004) 34:216–21. 10.1108/00346650410560398

[14] Ribas-Latre A Eckel-Mahan K. Interdependence of nutrient metabolism and the circadian clock system: importance for metabolic health. Mol Metab. (2016) 5:133–52. 10.1016/j.molmet.2015.12.006 26977390 PMC4770266

[15] Tsang AH Koch CE Kiehn J-T Schmidt CX Oster H. An adipokine feedback regulating diurnal food intake rhythms in mice. eLife. (2020) 9:e55388. 10.7554/eLife.55388 32644041 PMC7375813

[16] Ruddick-Collins LC Morgan PJ Johnstone AM. Mealtime: a circadian disruptor and determinant of energy balance? J Neuroendocrinol. (2020) 32:12886. 10.1111/jne.12886 32662577

[17] Blundell JE Gibbons C Caudwell P Finlayson G Hopkins M. Appetite control and energy balance: impact of exercise. Obes Rev. (2015) 16(Suppl. 1):67–76. 10.1111/obr.12257 25614205

[18] Li Y Schnabl K Gabler S-M Willershäuser M Reber J Karlas A. Secretin-activated brown fat mediates prandial thermogenesis to induce satiation. Cell. (2018) 175:1561–74.e12. 10.1016/j.cell.2018.10.016 30449620

[19] Shostak A Meyer-Kovac J Oster H. Circadian regulation of lipid mobilization in white adipose tissues. Diabetes. (2013) 62:2195–203. 10.2337/db12-1449 23434933 PMC3712056

[20] Riesenfeld G Sklan D Bar A Eisner U Hurwitz S. Glucose absorption and starch digestion in the intestine of the chicken. J Nutr. (1980) 110:117–21. 10.1093/jn/110.1.117 7354376

[21] Refinetti R. Circadian rhythmicity of body temperature and metabolism. Temperature. (2020) 7:321–62. 10.1080/23328940.2020.1743605 33251281 PMC7678948

[22] Gordon CJ. The mouse thermoregulatory system: its impact on translating biomedical data to humans. Physiol Behav. (2017) 179:55–66. 10.1016/j.physbeh.2017.05.026 28533176 PMC6196327

[23] Driver HS Shulman I Baker FC Buffenstein R. Energy content of the evening meal alters nocturnal body temperature but not sleep. Physiol Behav. (1999) 68:17–23. 10.1016/s0031-9384(99)00145-6 10627057

[24] Vinales KL Begaye B Thearle MS Krakoff J Piaggi P. Core body temperature, energy expenditure, and epinephrine during fasting, eucaloric feeding, and overfeeding in healthy adult men: evidence for a ceiling effect for human thermogenic response to diet. Metabolism. (2019) 94:59–68. 10.1016/j.metabol.2019.01.016 30710573 PMC6446552

[25] Hernández-González T González-Barrio R Escobar C Madrid JA Periago MJ Collado MC. Timing of chocolate intake affects hunger, substrate oxidation, and microbiota: a randomized controlled trial. FASEB J. (2021) 35:e21649. 10.1096/fj.202002770RR 34164846

[26] Kawamoto T Noshiro M Furukawa M Honda KK Nakashima A Ueshima T. Effects of fasting and re-feeding on the expression of Dec1, Per1, and other clock-related genes. J Biochem. (2006) 140:401–8. 10.1093/jb/mvj165 16873396

[27] Kmiec Z Pokrywka L Kotlarz G Kubasik J Szutowicz A Mysliwski A. Effects of fasting and refeeding on serum leptin, adiponectin and free fatty acid concentrations in young and old male rats. Gerontology. (2005) 51:357–62. 10.1159/000088698 16299415

[28] Haldar S Egli L De Castro CA Tay SL Koh MXN Darimont C. High or low glycemic index (GI) meals at dinner results in greater postprandial glycemia compared with breakfast: a randomized controlled trial. BMJ Open Diabetes Res Care. (2020) 8:e001099. 10.1136/bmjdrc-2019-001099 32327444 PMC7202752

[29] Leung GKW Huggins CE Bonham MP. Effect of meal timing on postprandial glucose responses to a low glycemic index meal: a crossover trial in healthy volunteers. Clin Nutr. (2019) 38:465–71. 10.1016/j.clnu.2017.11.010 29248250

[30] Saad A Dalla Man C Nandy DK Levine JA Bharucha AE Rizza RA. Diurnal pattern to insulin secretion and insulin action in healthy individuals. Diabetes. (2012) 61:2691–700. 10.2337/db11-1478 22751690 PMC3478548

[31] Sussman W Stevenson M Mowdawalla C Mota S Ragolia L Pan X. BMAL1 controls glucose uptake through paired-homeodomain transcription factor 4 in differentiated Caco-2 cells. Am J Physiol Cell Physiol. (2019) 317:C492–501. 10.1152/ajpcell.00058.2019 31216190 PMC6766619

[32] Iwashina I Mochizuki K Inamochi Y Goda T. Clock genes regulate the feeding schedule-dependent diurnal rhythm changes in hexose transporter gene expressions through the binding of BMAL1 to the promoter/enhancer and transcribed regions. J Nutr Biochem. (2011) 22:334–43. 10.1016/j.jnutbio.2010.02.012 20688499

