# Supplementary material for: Snack timing affects tissue clock and metabolic responses in male mice
Source: Front Nutr. 2022 Aug 11;9:956641. doi: 10.3389/fnut.2022.956641 (PMC9410699; doi:10.3389/fnut.2022.956641)
Supplement: Supplementary file 1 [file Data_Sheet_1.pdf]

Supplementary Material

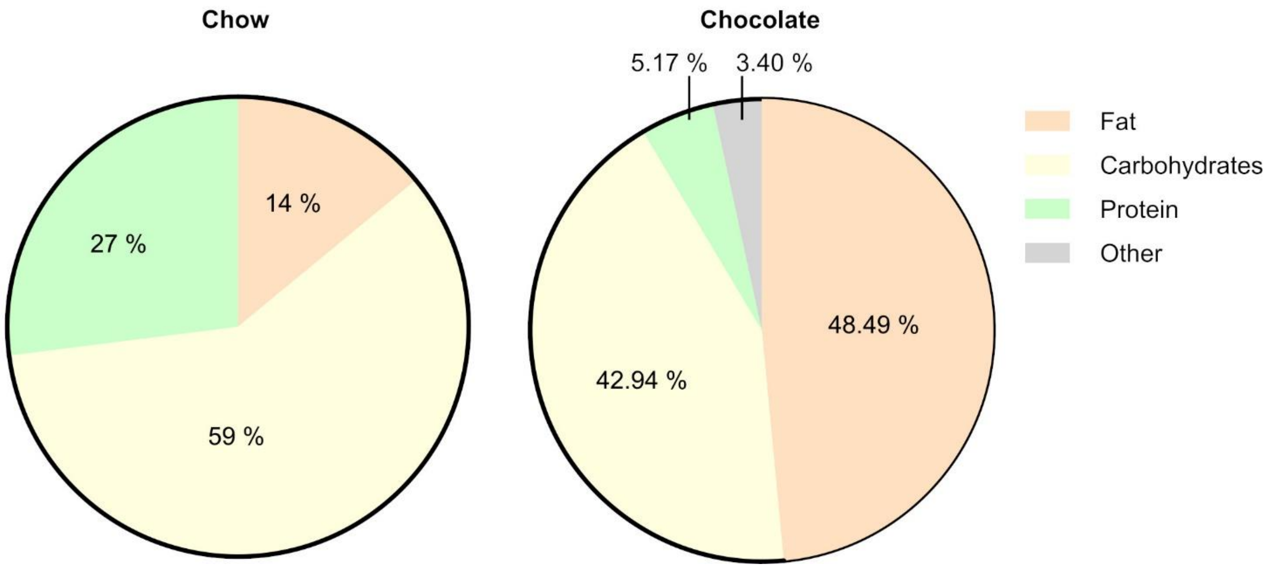

Supplementary Figure 1. Composition of chow and chocolate snacks.

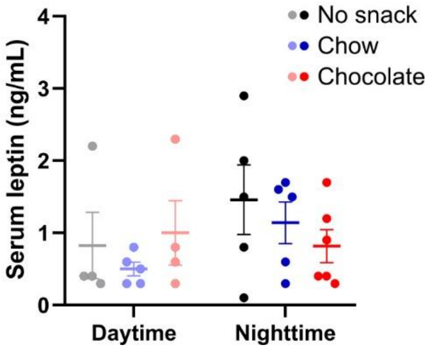

Supplementary Figure 2. Snacking does not alter serum leptin levels. Concentration of leptin in serum. Mice were fasted for 12 h and received no snack (control cohort), normal chow, or chocolate *ad libitum* for 20 min. Data are shown as mean ± SEM; n=4-6 per group; 2-way ANOVA: time, group, interaction $p>0.05$.

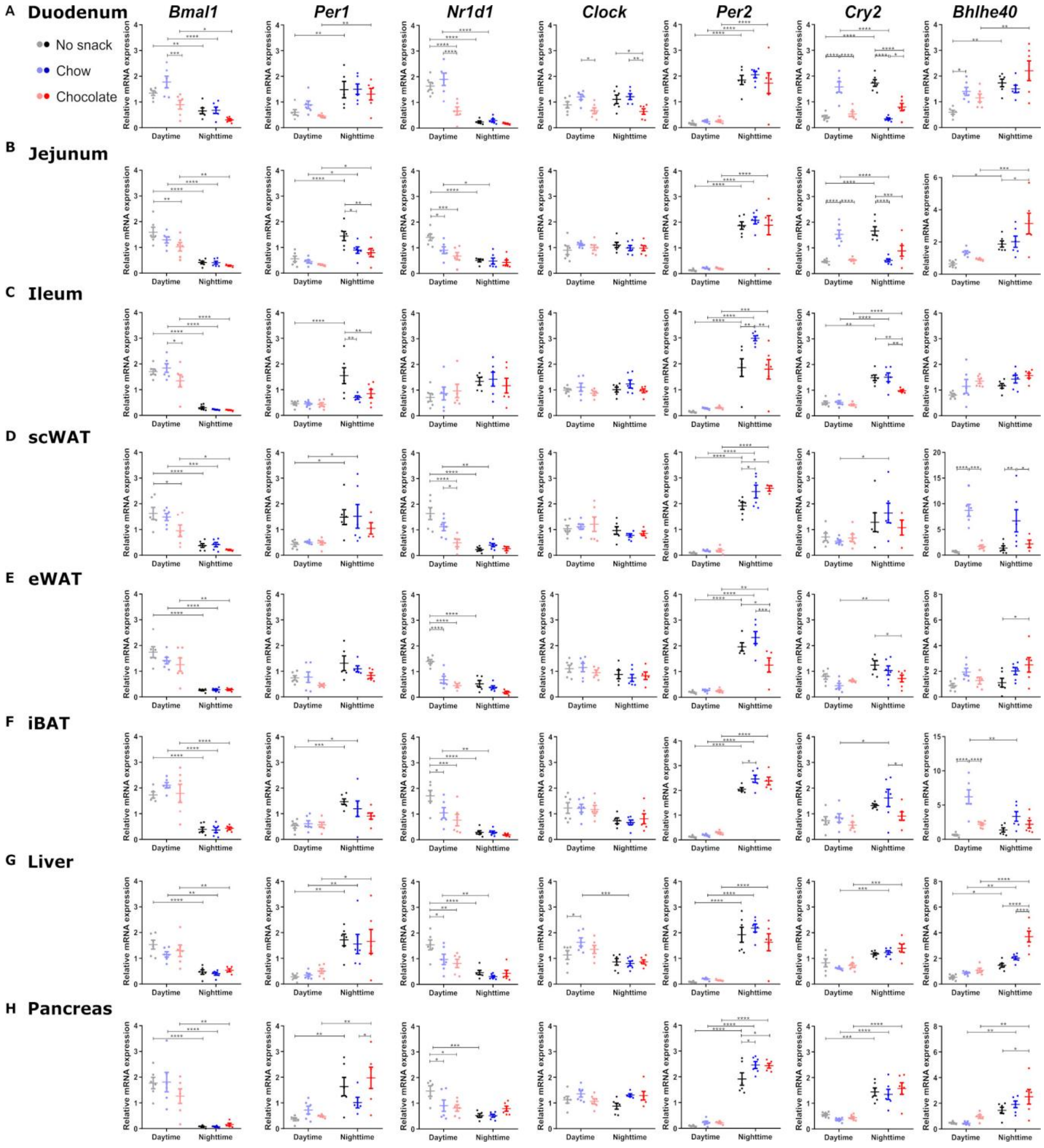

Supplementary Figure 3. Clock gene expression in peripheral tissues. Relative mRNA expression was determined for *Bmal1*, *Per1*, *Nr1d1*, *Clock*, *Per2*, *Cry2*, *Bhlhe40* in (A) duodenum, (B) jejunum, (C) ileum, (D) scWAT, (E) eWAT, (F) iBAT, (G) liver, and (H) pancreas. Data are shown as mean ± SEM; n=3-6 per group; Bonferroni post-hoc test: * $p<0.05$; ** $p<0.01$; *** $p<0.001$; **** $p<0.0001$; 2-way ANOVA: (A) *Bmal1* time $p<0.0001$, group $p<0.001$, interaction $p>0.05$, *Per1* time $p<0.0001$, group, interaction $p>0.05$, *Nr1d1* time $p<0.0001$, group $p<0.001$, interaction $p<0.01$, *Clock* time, interaction $p>0.05$, group $p<0.001$, *Per2* time $p<0.0001$, group, interaction $p>0.05$, *Cry2* time $p>0.05$, group $p<0.01$, interaction $p<0.0001$, *Bhlhe40* time $p<0.0001$, group, interaction $p<0.05$, (B)

Bmal1 time $p<0.0001$, group $p<0.05$, interaction $p>0.05$, *Per1* time $p<0.0001$, group $p<0.01$, interaction $p>0.05$, *Nr1d1* time $p<0.0001$, group $p<0.01$, interaction $p<0.05$, *Clock* time, group, interaction $p>0.05$, *Per2* time $p<0.0001$, group, interaction $p>0.05$, *Cry2* time $p>0.05$, group $p<0.05$, interaction $p<0.0001$, *Bhlhe40* time $p<0.0001$, group, interaction $p>0.05$, (C) *Bmal1* time $p<0.0001$, group, interaction $p>0.05$, *Per1* time $p<0.0001$, group, interaction $p<0.05$, *Nr1d1* time $p<0.05$, group, interaction $p>0.05$, *Clock* time, group, interaction $p>0.05$, *Per2* time $p<0.0001$, group, interaction $p<0.05$, *Cry2* time $p<0.0001$, group $p<0.01$, interaction $p>0.05$, *Bhlhe40* time, group $p<0.05$, interaction $p>0.05$, (D) *Bmal1* time $p<0.0001$, group, interaction $p>0.05$, *Per1* time $p<0.001$, group, interaction $p>0.05$, *Nr1d1* time $p<0.0001$, group, interaction $p<0.01$, *Clock* time, group, interaction $p>0.05$, *Per2* time $p<0.0001$, group $p<0.05$, interaction $p>0.05$, *Cry2* time $p<0.01$, group, interaction $p>0.05$, *Bhlhe40* time $p>0.05$, group $p<0.0001$, interaction $p>0.05$, (E) *Bmal1* time $p<0.0001$, group, interaction $p>0.05$, *Per1* time $p<0.01$, group, interaction $p>0.05$, *Nr1d1* time, group $p<0.0001$, interaction $p<0.01$, *Clock* time $p<0.05$, group, interaction $p>0.05$, *Per2* time $p<0.0001$, group, interaction $p<0.05$, *Cry2* time $p<0.01$, group $p<0.05$, interaction $p>0.05$, *Bhlhe40* time $p>0.05$, group $p<0.05$, interaction $p>0.05$, (F) *Bmal1* time $p<0.0001$, group, interaction $p>0.05$, *Per1* time $p<0.0001$, group, interaction $p>0.05$, *Nr1d1* time $p<0.0001$, group $p<0.01$, interaction $p<0.05$, *Clock* time $p<0.01$, group, interaction $p>0.05$, *Per2* time $p<0.0001$, group $p<0.05$, interaction $p>0.05$, *Cry2* time $p<0.01$, group, interaction $p>0.05$, *Bhlhe40* time $p>0.05$, group $p<00001$, interaction $p<0.01$, (G) *Bmal1* time $p<0.0001$, group, interaction $p>0.05$, *Per1* time $p<0.0001$, group, interaction $p>0.05$, *Nr1d1* time $p<0.0001$, group $p<0.05$, interaction $p>0.05$, *Clock* time $p<0.0001$, group, interaction, $p>0.05$, *Per2* time $p<0.0001$, group, interaction $p>0.05$, *Cry2* time $p<0.0001$, group, interaction $p>0.05$, *Bhlhe40* time, group $p<0.0001$, interaction $p<0.001$, (H) *Bmal1* time $p<0.0001$, group, interaction $p>0.05$, *Per1* time $p<0.0001$, group, interaction $p>0.05$, *Nr1d1* time $p<0.001$, group $p>0.05$, interaction $p<0.05$, *Clock* time, interaction $p>0.05$, group $p<0.05$, *Per2* time $p<0.0001$, group $p<0.05$, interaction $p>0.05$, *Cry2* time $p<0.0001$, group, interaction $p>0.05$, *Bhlhe40* $p<0.0001$, group $p<0.05$, interaction $p>0.05$.

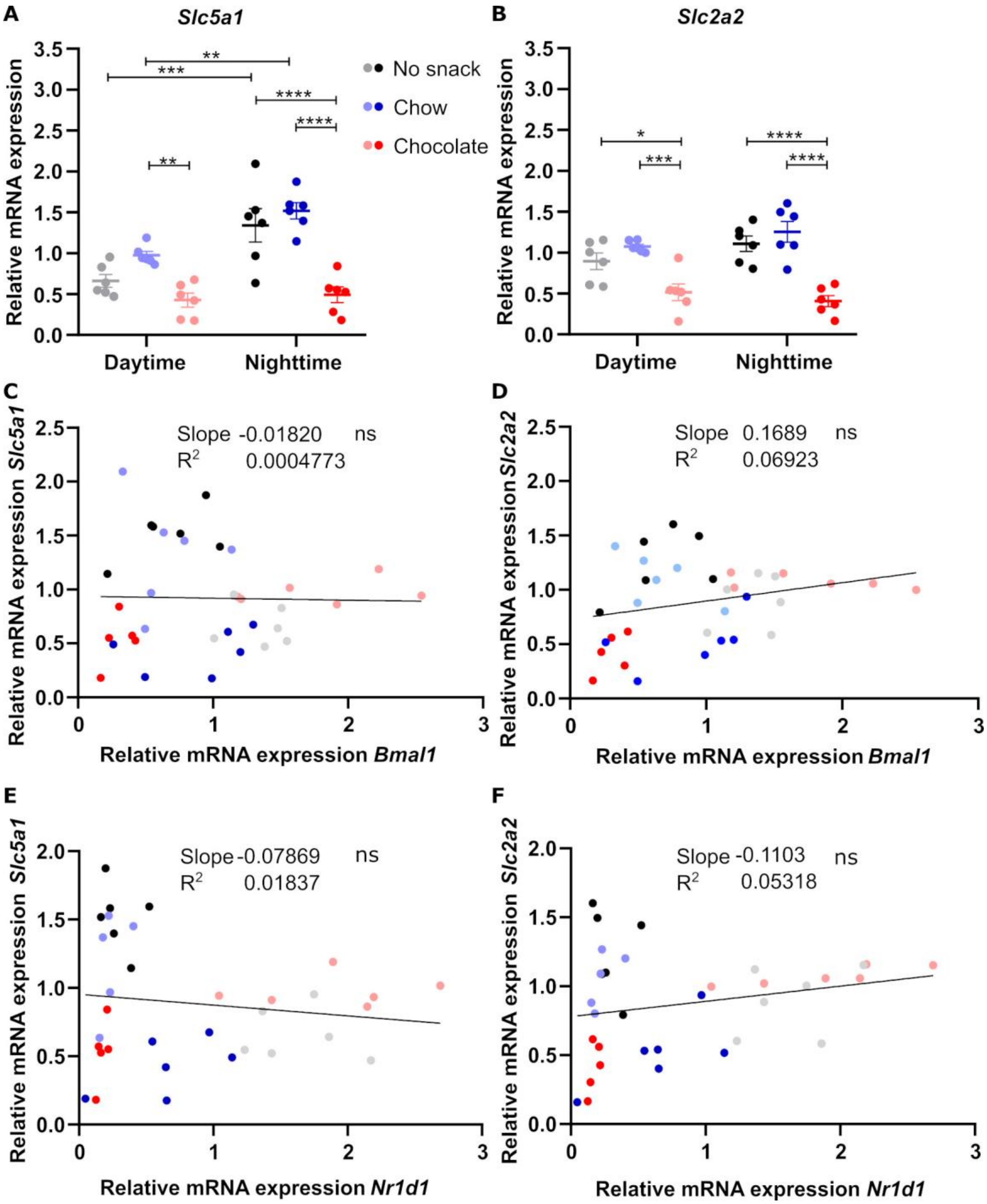

Supplementary Figure 4. Duodenal glucose uptake transporter gene expression is influenced by snack type but does not correlate with clock gene expression. Relative mRNA expression of (A) *Slc5a1*, (B) *Slc2a2*. Linear regression of (C) *Bmal1* and *Slc5a1*, (D) *Bmal1* and *Slc2a2*, (E) *Nr1d1* and *Slc5a1*, (F) *Nr1d1* and *Slc2a2*. (A,B) Data are shown as mean ± SEM; n=5-6 per group; Bonferroni post-hoc test: * $p<0.05$; ** $p<0.01$; *** $p<0.001$; **** $p<0.0001$; 2-way ANOVA: (A) time, group $p<0.0001$, interaction $p<0.05$, (B) time, interaction $p>0.05$, group $p<0.0001$. (C-F) Simple linear regression analysis; n=5-6 per group.
